# Supplementary material for: Feasibility, acceptability, and effectiveness of group antenatal care on the continuum of care and perinatal outcomes in Sub-Saharan Africa: A systematic review and meta-analysis protocol
Source: PLoS One. 2025 Apr 22;20(4):e0311473. doi: 10.1371/journal.pone.0311473 (PMC12013882; doi:10.1371/journal.pone.0311473)
Supplement: Attachment — Submitted filename: Response to reviewers.pdf [file pone.0311473.s004.pdf]

<u>**Date: February 13, 2025.**</u>

Dear Editor-in-chief,

Subject: <u>Submission of the corrected systematic review and meta-analysis protocol manuscript.</u>

We wish to extend our gratitude to the editor and reviewers for their valuable and constructive comments and suggestions. Please find attached the author's point-by-point response letter addressing the editor's and reviewers' comments and suggestions. Also attached are a clean protocol manuscript and the tracked changes document for the systematic review and meta-analysis protocol entitled <u>**"Feasibility, Acceptability, and Effectiveness of Group Antenatal Care on the Continuum of Care and Perinatal Outcomes in Sub-Saharan Africa: A Systematic Review and Meta-analysis Protocol"**</u> with manuscript number PONE-D-24-40365 by Mengesha MB, et al., for your consideration.

We thoroughly revised the systematic review and meta-analysis protocol in response to the editor's and reviewers' comments and suggestions. We confidently assert that the revised protocol meets the publication standards of your esteemed journal. We look forward to your response, appreciate your consideration, and await the outcome of your assessment.

Yours sincerely,

Meresa

Meresa Berwo Mengesha (BSc, MSc, Assistant Professor), Corresponding Author
Department of Midwifery, College of Medicine and Health Sciences,
Adigrat University, Adigrat, Ethiopia.
Email: meresaberu@gmail.com.

Response to the Academic Editor's and reviewer's comments

Additional Editor Comments

Introduction:

1. **Please restructure this section.** Start with broader topics such as maternal mortality - globally and the burden in SSA. Solicit the link in between maternal and perinatal mortality with ANC. Define both ANC and G-ANC. Then discuss the gap - GANC, then discuss what you are going to do about it - in this case systematic review and meta-analysis.

Author's response: Dear academic editor, thank you very much for your valuable recommendation to create a catchy and structured outline for the introduction; we appreciate your comments and have revised it according to the suggested ideas.

2. **Line 59 "pregnancy and perinatal outcome**" - please revise this terminology. Perinatal outcome is a pregnancy outcome. Possible suggestion - maternal and perinatal outcome.

   Author's response: Thank you very much for your valuable recommendation to revise this terminology. It has been modified as per your recommendation.

3. **Line 64 - 66:** "This model is also shown to benefit marginalized women, whose perinatal outcomes are comparable to those in developed countries (8, 9)." This is a confusing statement. Do you mean that the studies show that having GANC brings perinatal outcomes to the same level to those in developed countries? Please rewrite it.

   Author's response: Thank you for seeking clarification on this issue. "This model is also shown to benefit marginalized women, whose perinatal outcomes are comparable to those in developed countries." We mean that the findings indicate a comprehensive, women-centered group antenatal care intervention provided to disadvantaged women may lead to maternal and perinatal outcomes that are relatively comparable to those in developed countries. However, this does not necessarily imply the same level as in developed nations, as there are other factors to consider. It has been revised so that "relatively comparable" appears in the protocol with the tracked changes.

4. **Line 93 - "this review" - this is not a review yet**. this is a protocol. Please review throughout the manuscript as a protocol not as a review.

   Author's response: Thank you for the recommendation. We have revised it according to the suggested idea. It is now titled "systematic review and meta-analysis protocol."

Methods section:

5. **Population -** are you going to select vulnerable population in the Sub-Saharan Africa or you are defining women in Sub-Saharan Africa? The way you described your population is confusing.

   Author's response: Thank you for raising this critical issue. To clarify any confusion regarding the selection of the target population, we plan to include population groups primarily focused on pregnant women—whether in their first or subsequent pregnancies—who are receiving antenatal care. This will encompass adolescents and young women, women from rural areas, women with low socioeconomic status, women with limited education, HIV-positive women, multiparous and primiparous women, women from diverse ethnic and cultural backgrounds, and women with high-risk pregnancies. Additionally, we will consider healthcare providers, community health workers, and traditional birth attendants, who play essential roles in facilitating group antenatal care. This information is added to the population portions of the subheading under the methods section. Please review the manuscript with track changes.

6. How is feasibility your secondary outcome? "Feasibility, Acceptability, and Effectiveness of Group Antenatal Care on the Continuum of Care and Perinatal Outcomes in Sub-Saharan Africa: A Systematic Review and Meta-Analysis Protocol" This is your research title. How can your primary objective be a secondary outcome? Your primary objective should be to assess feasibility, acceptability, and effectiveness.

   Author's response: Thank you for raising this concern. We have revised it, and the following assertions have been added to the protocol with the track changes: "The primary outcomes will be the effect of G-ANC on attendance at four or more antenatal care visits, utilization of institutional delivery, impact on perinatal outcomes (birth weight, preterm birth, neonatal intensive care unit admission, gestational age), attendance at postnatal care visits, uptake of any postpartum family planning methods, and the acceptability and feasibility of this model of care by pregnant women, healthcare providers, and community health workers."

Secondary outcomes

Effect of group ANC/PNC on the Quality of care outcomes, maternal health outcomes (uptake of healthy behaviors, increased health literacy and self-efficacy and other maternal health services), Behavioral and social outcomes, cultural sensitivity and acceptability issues will be considered as secondary outcomes.”

7. The way you selected the study period sounds random. Why did you choose from 2016 to mid-2024? Why do you want to examine G-ANC in this time period?

Author’s response: We appreciate your thoughtful insight regarding the time restriction of this review. Why was the study period limited to certain years (in this case, from January 1, 2016, to June 30, 2024)? Certainly, it is confined to these years for the following reasons.

1. Since the initiation of research in this area (in 2016, the Global Group Antenatal Care Collaborative was established as a platform for group antenatal care researchers working in low- and middle-income countries to share experiences and shape future studies to enhance the development of a solid global evidence base reflecting implementation and outcomes specific to low- and middle-income countries), most studies in this specific field within LMICs have been conducted after 2016. You can read this article (Building a Global Evidence Base to Guide Policy and Implementation for Group Antenatal Care in Low- and Middle-Income Countries: Key Principles and Research Framework Recommendations from the Global Group Antenatal Care Collaborative) (escholarship.org).
2. Around this time, after 2016, the World Health Organization (WHO) and other global health organizations began to emphasize the importance of person-centered care in maternal health, marking a shift toward this approach. Group ANC, which promotes peer support and active participation, aligns well with this model, prompting further research into its feasibility and effectiveness.
3. After 2015, the Sustainable Development Goals (SDGs) were launched, with SDG 3 specifically targeting maternal and child health. This led to increased funding and support for maternal health innovations, including group ANC, from global health organizations, governments, and NGOs. We aimed to see the effectiveness of this model in this case.

4. Furthermore, the release of updated WHO guidelines on antenatal care in 2016, which recommended a minimum of eight ANC contacts and emphasized the importance of quality care, provided a strong impetus for exploring innovative models like group ANC. Overall, the increase in studies on group ANC in LMICs after 2016 restricted our ability to assess the model's feasibility, acceptability, and effectiveness in low-resource settings.

8. **Your exclusion needs revision.** There won't be a case report or case series written on G-ANC - not even a chance.

   Author's response: Thank you for highlighting this intriguing point. While case reports and case series are not typically used in GANC research, they could be valuable in documenting unique implementations and challenges in low-resource settings. However, to date, most existing studies have utilized larger-scale designs, such as randomized controlled trials (RCTs), mixed-methods studies, and quasi-experimental designs, to evaluate the effectiveness and feasibility of GANC. We considered including these less common designs if they offered meaningful insights into the implementation and challenges of this care model. However, after a thorough search and screening process during the review, we found no such studies. As a result, we have removed the statement regarding "case reports and case series" and updated the protocol accordingly.

9. **Search Strategy**: This section is written as if you have already conducted the systematic review and meta-analysis. Please revise it.

   Author's response: Thank you for your request to revise this section. We have made the necessary revisions, and we plan to address and update it at the end of the review process.

Responses to Reviewer 1:

I thank the Editor for letting me review the paper. The research question is fundamental, and the protocol is suitable overall.

Author's response: Dear reviewer, we are grateful for your time and significant contributions to this protocol. We learned a great deal from your scientific arguments.

Abstract

1. You have failed to acknowledge that this is a protocol; you have structured your abstract as if you have conducted the systemic review and meta-analysis. You are advised to revise the aim statement of your abstract.

Author's response: Thank you for your critical observation and for informing us about this. In our context, a planned systematic review and meta-analysis refers to the systematic review and meta-analysis protocol. We have revised it according to your recommendation. Please take a look at the revised protocol with track changes.

2. Please use 'the planned review 'or 'the review' when addressing the systemic review and meta-analysis. Use this protocol when you are addressing the protocol.

Author's response: We have amended it according to the suggestion given for improvement.

Introduction

1. Please begin your paragraph with the burden of maternal mortality and perinatal mortality. Then, provide evidence that ANC is one of the core strategies for mitigating the burden. Then, in subsequent paragraphs, you argue the importance of G ANC VS ANC, geographic disparity, the paucity of meta-analysis and systemic review, and the need to conduct the study.

Author's response: Thank you very much for your valuable recommendation to create a catchy and structured outline of the introduction section. We appreciate your feedback and have revised the document accordingly. Please take a look at the revised protocol with the tracked changes.

2. Again, please revise your aim statement.

   Author's response: we accept the comments and we made revisions as per the suggested idea. Kindly have a look at the revised protocol with the track changes.

Methods

Primary outcomes

1. Maternal morbidity and mortality are not typically considered as 'perinatal outcomes.' Perinatal outcome refers to the status of the fetus or newborn during the perinatal period, which is between 24-28, depending on the setup, up to seven days after birth. Usually utilized perinatal outcomes include birthweight, preterm birth, neonatal intensive care unit admission, gestational age, stillbirth, early neonatal death, and neonatal morbidities. Therefore, you should revise your statements accordingly and avoid conflating maternal and perinatal outcomes.

   Author's response: We appreciate your critical observation and admit that it was an editorial error. It is revised in the manuscript with the track changes according to the suggested idea.

2. Please reconcile what you wrote in the 'Outcomes and prioritization 'section with primary and secondary outcomes. Furthermore, you need to be specific.

   Author's response: We have checked it and revised it accordingly. The outcome and prioritization align with the above-specified primary and secondary outcomes. Please kindly have a look at the revised protocol with the track changes.

3. **Please describe the intervention group instead of saying 'G-ANC and/or Participatory Group.'**

   Author's response: Thank you for reminding us to include the intervention description (G-ANC). The statement provided below has also been added to the protocol with track changes.

The intervention is G-ANC, which is defined as the integration of conventional antenatal assessments with group discussions and support. Typically involves 8 to 12 women who are at similar gestational ages of pregnancy, guided by 1 to 2 group leaders. These leaders adopt a highly participatory and facilitative approach during the G-ANC sessions. The number of sessions can be adjusted to align with both global and local guidelines regarding the required frequency of visits. Each session is structured to include clinical care, client education, and ongoing discussions. During these meetings, healthcare providers deliver clinical care, and participants engage in self-assessments, such as monitoring their blood pressure and weight or identifying any warning signs. These group meetings count as antenatal care visits. The same group of women and facilitators attends all session's together, fostering stability, trust, and a sense of community among the participants.

Inclusion / Exclusion criteria

1. Could you please clarify whether you will include published abstracts, conference proceedings, and reference lists of the studies that are incorporated?

   Author's response: We will include published articles written in English. Additionally, we will incorporate the reference lists of studies by applying both retrospective reference harvesting and prospective forward citation searching techniques. However, we will exclude preprints that have not been extensively reviewed, unpublished reviews, conference proceedings, commentaries, editor's letters, and non-English publications.

Search strategies

1. Please improve the grammar of the paragraph; mix of tenses.

   Author's response: Thank you for your request to revise this section. We have made the necessary revisions, as we intend to address them and update them after the review process.

Risk of bias assessment

1. Please mention the techniques that will be used to mitigate the JBI critical appraisal tools. Such as training….

Author's response: Thank you for your recommendation to add strategies for mitigating the limitations of the JBI critical appraisal tools in the systematic review and meta-analysis. Following your suggestion, the following statements were added to the protocol with track changes: "To address the limitations of using the Joanna Briggs Institute (JBI) critical appraisal tools, the following techniques will be employed to ensure the quality and reliability of the appraisal process. Comprehensive training will be provided for reviewers on effectively using the JBI critical appraisal tools. Additionally, standardized and clear guidelines will be utilized to ensure consistency across reviewers for each study design. Moreover, we will conduct pilot appraisals on a small subset of studies to identify potential challenges or ambiguities in the tool's application. Furthermore, we will recruit three independent reviewers to appraise studies and compare results to minimize bias. Finally, clear documentation of the decisions made during the appraisal process will enhance transparency and reproducibility."

Reviewer 2

General comment;

1. It is well-written and very important for the area.

   Author's response: Thank you very much for your kind words and positive feedback on our work. We are delighted to hear that you found our protocol valuable and well-presented. We learned a lot from your scientific arguments.

Specific comment:

Abstract:

2. Line number 32-24: The justification for the importance of this systematic review and meta-analysis needs to be strengthened. The author mentions that 'pilot studies show promising evidence of its effectiveness in these areas.' To enhance this rationale, it is crucial to highlight additional factors, such as conflicting findings in existing literature and unresolved research gaps. This will help better identify existing gaps and provide stronger justification for assessing the outcomes.

   Author's response: Thank you for your critical insight on this issue. To enhance the aim and rationale of this review, we have added additional factors such as the insufficient evidence regarding its effectiveness in reducing maternal and perinatal mortality in the region. Furthermore, the disparity in maternal mortality rates between developing and developed countries highlights the pressing need for targeted interventions. Additionally, there is a lack of comprehensive meta-analyses and systematic reviews evaluating GANC's effectiveness across various settings. Current studies exhibit considerable variability, emphasizing the need to consolidate findings from multiple research efforts to provide a clearer understanding of the overall effectiveness of group antenatal interventions. Kindly have a look at page 4, line numbers 103-111 in the protocol with the track changes.

2. **Line number 47-48:** In the abstract method section, it states, "Pre-specified subgroup analysis and sensitivity analysis will be conducted as necessary." If there is heterogeneity, subgroup analysis is recommended based on scientific evidence. Therefore, why it stated as necessary.

Author's response: Thank you for seeking clarification on this issue. Subgroup analyses and sensitivity analyses are typically performed when there is evidence of heterogeneity (e.g., statistical or clinical) in the results. We pre-specified subgroup and sensitivity analyses in our protocol to ensure transparency and scientific rigor. However, these analyses will only be conducted if heterogeneity or other scientific evidence indicates they are necessary. This approach ensures that we avoid unnecessary multiple testing and concentrate on meaningful, data-driven explorations.

Methods

3. **Lines 112-117:** The primary outcomes are numerous. It would be beneficial to narrow them down to a few specific, measurable outcomes that clearly define the effect measures.

   Author's response: Thank you very much for your valuable recommendation for specific and measurable outcomes that clearly define the effect measures. We have included the following items in the revised protocol, tracked in changes: "The primary outcomes will be the effect of G-ANC on attendance at four or more antenatal care visits, utilization of institutional delivery, impact on perinatal outcomes (birth weight, preterm birth, neonatal intensive care unit admissions, gestational age), attendance at postnatal care visits, uptake of any postpartum family planning methods, and the acceptability and feasibility of this model of care by pregnant women, healthcare providers, and community health workers."

4. **Line 122-123: Setting:** it will be focused on Health facility type, rural and urban setting? Additionally, GANC expected to be effective only in rural areas or in communities where health facilities are not well implemented and accessible.

   Author's response: We appreciate your critical observations and expert opinions regarding the settings of studies conducted on group antenatal care. In response to your recommendation, we have added the following specific details to the protocol, as tracked in the revised document.

   Group antenatal care (GANC) has been studied in various settings across Sub-Saharan Africa, with a particular focus on low-resource environments where maternal health outcomes are often suboptimal. This study will include rural, urban, and peri-urban areas and various health facility types, such as primary health centers, health posts, and community-based clinics.

5. Lines 129-130: 'This review will exclude preprints, unpublished reviews, case reports, case series, commentaries, editor's letters, and non-English publications.' One of the key principles of systematic reviews and meta-analyses is to capture both positive and negative findings, as negative results are often underreported in published literature. Since many studies, including those from pharmaceutical companies, may not publish negative findings, incorporating grey literature can strengthen the systematic review and meta-analysis by reducing publication bias.

Author's response: Thank you for seeking clarification on this crucial issue of publication bias. While we recognize the value of grey literature—such as preprints, unpublished studies, case reports, case series, commentaries, editor letters, and non-English sources—in addressing publication bias and capturing unreported negative findings, we have excluded these materials from our review. This decision aims to uphold a consistent level of methodological rigor and reliability. Peer-reviewed publications undergo thorough evaluation, which enhances the credibility and reproducibility of their findings.

To address potential publication bias, we will implement a comprehensive search strategy across multiple databases, including PubMed/MEDLINE, Web of Science, EMBASE, and CINAHL, to capture both positive and negative results. We will also incorporate registered clinical trials (e.g., from ClinicalTrials.gov) and thoroughly review ProQuest repositories from institutions that meet our inclusion criteria.

In our meta-analysis, we will evaluate and adjust for publication bias using statistical methods such as funnel plots, Egger's test, and trim-and-fill analysis. We will interpret our findings cautiously in light of any identified bias. We understand that excluding grey literature may restrict our ability to capture all relevant studies, particularly those with negative findings that remain unpublished. This could introduce some publication bias, and we will interpret our results with this limitation in mind. In summary, while we acknowledge the potential benefits of including grey literature, we excluded it because of the need for methodological consistency and reliability. We will implement strategies to minimize publication bias through a thorough search strategy, including registered trials and statistical evaluations.

6. Lines 138-139: The search strategy needs to be clearer. In addition to using MeSH terms, it would be beneficial to explain whether a framework such as PICO, SPICE, or SPIDER is used, especially since the inclusion criteria cover qualitative and mixed-method studies.

Author's response: Thank you very much for your critical insights on this issue. Besides the Medical Subject Headings (MeSH) terms, this systematic review and meta-analysis protocol will utilize the PICO framework (Population, Intervention, Comparator, Outcome). This method is particularly fitting for our research, which examines the impact of Group Antenatal Care (G-ANC) on maternal and perinatal outcomes in low-resource settings. We will apply the PICO framework in our review protocol as follows: Population: This will focus on expectant mothers, with a particular emphasis on those in resource-limited environments or from disadvantaged groups; Intervention: The intervention is G-ANC, defined as the integration of traditional antenatal assessments with group discussions and support. Comparator: We will compare G-ANC with individualized, standard, conventional, or traditional models of care. Outcome: Primary outcomes of interest will include feasibility, perinatal outcomes, and utilization of maternal health care services/continuum of maternal health care, while other reported outcome events may be considered secondary outcomes.

7**. Lines 218-219**: "Or if the data is incomplete, a qualitative synthesis will be conducted instead of a meta-analysis." It would be beneficial to recommend additional analytical approaches, such as subgroup analysis, sensitivity analysis, or meta-regression, rather than solely opting for qualitative synthesis. Additionally, considering the risk of bias, studies with certain designs may be excluded to improve the robustness of the analysis.

Author's response: Thank you for your valuable suggestion; we revised it based on your idea.
