# Supplementary material for: Feasibility, acceptability, and effectiveness of group antenatal care on the continuum of care and perinatal outcomes in Sub-Saharan Africa: A systematic review and meta-analysis protocol
Source: PLoS One. 2025 Apr 22;20(4):e0311473. doi: 10.1371/journal.pone.0311473 (PMC12013882; doi:10.1371/journal.pone.0311473)
Supplement: Attachment — Submitted filename: Systemic review meta analysis review Protocol_5_1_2025.docx [file pone.0311473.s002.docx]

I thank the Editor for letting me review the paper.

The research question is fundamental, and the protocol is suitable overall.

Abstract

You have failed to acknowledge that this is a protocol; you have structured your abstract as if you have conducted the systemic review and meta-analysis. You are advised to revise the aim statement of your abstract.

Please use ‘the planned review ‘ or  ‘the review’ when addressing the systemic review and meta-analysis. Use this protocol when you are addressing the protocol.

Introduction

Please begin your paragraph with the burden of maternal mortality and perinatal mortality. Then, provide evidence that ANC is one of the core strategies for mitigating the burden. Then, in subsequent paragraphs, you argue the importance of G ANC VS ANC, geographic disparity, the paucity of meta-analysis and systemic review, and the need to conduct the study.

Again, please revise your aim statement.

Methods

Primary outcomes

Maternal morbidity and mortality are not typically considered as ‘perinatal outcomes.’ Perinatal outcome refers to the status of the fetus or newborn during the perinatal period, which is between 24-28, depending on the setup, up to seven days after birth. Usually utilized perinatal outcomes include birthweight, preterm birth, neonatal intensive care unit admission, gestational age, stillbirth, early neonatal death, and neonatal morbidities. Therefore, you should revise your statements accordingly and avoid conflating maternal and perinatal outcomes.

Please reconcile what you wrote in the  ‘ Outcomes and prioritization ‘ section with primary and secondary outcomes. Furthermore, you need to be specific.

Please describe the intervention group instead of saying ‘G-ANC and/or Participatory Group.’

Inclusion / Exclusion criteria

Could you please clarify whether you will include published abstracts, conference proceedings, and reference lists of the studies that are incorporated?

Search strategies

Please improve the grammar of the paragraph; mix of tenses.

Risk of bias assessment

Please mention the techniques that will be used to mitigate the JBI critical appraisal tools. Such as training….
