# Supplementary material for: Feasibility, acceptability, and effectiveness of group antenatal care on the continuum of care and perinatal outcomes in Sub-Saharan Africa: A systematic review and meta-analysis protocol
Source: PLoS One. 2025 Apr 22;20(4):e0311473. doi: 10.1371/journal.pone.0311473 (PMC12013882; doi:10.1371/journal.pone.0311473)
Supplement: Attachment — Submitted filename: Point_by_point_response_to_reviewers_auresp_3.docx [file pone.0311473.s006.docx]

**Date: February 27, 2025.**

**Dear Editor-in-chief,**

**Subject: Submission of the corrected systematic review and meta-analysis protocol manuscript.**

We wish to extend our gratitude to the editor and reviewers for their valuable and constructive comments and suggestions. Please find attached the author’s point-by-point response letter addressing the editor’s and reviewers’ comments and suggestions. Also attached are a clean protocol manuscript and the tracked changes document for the systematic review and meta-analysis protocol entitled **“Feasibility, Acceptability, and Effectiveness of Group Antenatal Care on the Continuum of Care and Perinatal Outcomes in Sub-Saharan Africa: A Systematic Review and Meta-analysis Protocol”** with manuscript ID PONE-D-24-40365 by Mengesha MB, et al., for your consideration.

We thoroughly revised the systematic review and meta-analysis protocol in response to the editor’s and reviewers’ comments and suggestions. We confidently assert that the revised protocol meets the publication standards of your esteemed journal. We look forward to your response, appreciate your consideration, and await the outcome of your assessment.

**Yours sincerely,**

**Meresa**

**Meresa Berwo Mengesha (BSc, MSc, Assistant Professor), Corresponding Author**

Department of Midwifery, College of Medicine and Health Sciences,

Adigrat University, Adigrat, Ethiopia.

Email: [meresaberu@gmail.com](mailto:meresaberu@gmail.com).

**Response to reviewers and editor’s comments**

**Journal Requirements:**

Please review your reference list to ensure that it is complete and correct. If you have cited papers that have been retracted, please include the rationale for doing so in the manuscript text, or remove these references and replace them with relevant current references. Any changes to the reference list should be mentioned in the rebuttal letter that accompanies your revised manuscript. If you need to cite a retracted article, indicate the article’s retracted status in the References list and also include a citation and full reference for the retraction notice.

**Author’s response:** Thank you for your critical observation and for bringing this to our attention. We have cross-checked the references twice and confirmed that we have not cited any retracted papers. However, we have made revisions to address some instances of duplication and ensured that the reference is now complete and accurate.

**Additional Editor Comments:**

Line 64 - 66: "This model is also shown to benefit marginalized women; whose perinatal outcomes are comparable to those in developed countries". While your descriptive response was good, the actual revision you made is still confusing. Please revise this statement.

**Author’s response:** Dear editor**,** thank you for your feedback and seeking clarification on this issue. We have revised it accordingly. The statement provided below has also been added to the protocol with track changes. “This model of care also benefits women from marginalized populations who experience maternal and perinatal outcomes comparable to those observed in certain low- and middle-income countries” Please take a look at the revised protocol with the tracked changes.
